# Targeted Induction of Cancer Cell Necroptosis Potentiates Anti-PD-1 Immunotherapy via CD80 Activation

**DOI:** 10.7150/ijbs.121690

**Published:** 2026-01-22

**Authors:** Xu Zhang, Detian Zhang, Zhe Zhou, Waner Liu, Susi Zhu, Siyu Xiong, Xiang Chen, Cong Peng

**Affiliations:** 1 The Department of Dermatology, Xiangya Hospital, Central South University, Changsha, Hunan 410008, China.; 2 Furong Labratory, Changsha, Hunan 410028, China.; 3 Hunan Key Laboratory of Skin Cancer and Psoriasis, Xiangya Hospital, Central South University, Changsha, Hunan 410008, China.; 4 Hunan Engineering Research Center of Skin Health and Disease, Xiangya Hospital, Central South University, Changsha, Hunan 410008, China.; 5 National Clinical Research Center for Geriatric Disorders, Xiangya Hospital, Central South University, Changsha, Hunan 410008, China.; 6 Key Laboratory of Traditional Chinese Medicine Syndrome / National Institute of Extremely-Weak Magnetic Field Infrastructure, Hangzhou 310028, China.

**Keywords:** necroptosis, tumor immune microenvironment, immunotherapy, TRADD, CD80

## Abstract

Insufficient infiltration or dysfunction of lymphocytes in the tumor immune microenvironment is considered to be a contributing factor to poor immunotherapy outcomes in solid tumors. Necroptosis, a form of immunogenic cell death, has attracted increasing interest because of its unique role in regulating tumor immune responses. CL-387785, a third-generation EGFR inhibitor, has been reported to inhibit tumors by regulating the cell cycle and inducing apoptosis; however, the underlying mechanisms remain unclear. In this study, we demonstrated that CL-387785 effectively suppressed the malignant phenotype of melanoma and lung cancer and confirmed that cancer cells undergo necroptosis, as evidenced by morphological and protein-level analyses. Further in vivo and in vitro experiments revealed that CL-387785 enhances tumor cell killing by immune cells by inducing CD80 expression on the tumor cell surface, thereby increasing CD8^+^ T lymphocyte function. Detailed mechanistic studies indicated that CL-387785 targets TRADD, recruiting RIPK1 to induce necroptosis in tumor cells, with subsequent nuclear translocation of NF-κB, which regulates CD80 transcription. In conclusion, our findings indicate that CL-387785 induces necroptosis in tumor cells via the TRADD/RIPK1/NF-κB/CD80 signaling pathway, thereby sensitizing tumors to anti-PD-1 therapy. These results suggest that CL-387785 is a promising candidate for increasing tumor immunotherapy efficacy.

## 1. Introduction

Cancer is a major global health problem, with approximately 10 million individuals dying annually worldwide^1^. Recently, immunotherapy options, such as monoclonal antibodies targeting PD-1 (nivolumab and pembrolizumab) and CTLA-4 inhibitors (ipilimumab), have yielded promising outcomes for melanoma and lung cancer^2^-^4^. Despite the favorable therapeutic outcomes of the above treatments, targeted therapy alone results in a limited progression-free survival period of only 5.3 months^5^. Patients receiving immunotherapy are susceptible to rapid development of drug resistance, and an abnormal immune microenvironment accounts for a large portion of primary and secondary resistance to anti-PD-1 mAbs. Moreover, certain signals in the microenvironment can induce cytotoxic T-cell (CTL) consumption, and sustained antigen stimulation can even induce T-cell alteration, eventually leading to immune desensitization of tumor cells and loss of T-cell function^6^-^8^. Therefore, the development of effective combination therapies to sensitize tumor cells is urgent for immunotherapy.

Immunogenic cell death (ICD) is a type of regulatory cell death triggered by chemotherapeutic drugs, natural compounds or other treatments to activate long-lasting antitumor immune responses in immunocompetent individuals^9^-^11^. Necroptosis, owing to its ability to release damage-associated molecular pattern (DAMP) molecules and activate T-cell immune responses, is also classified within the family of ICDs, along with ferroptosis and pyroptosis^12^. These released DAMPs act as 'eat me' or 'find me' signals by translocating to the cell membrane surface or spreading around the tumor tissue, facilitating the identification of tumor cells by antigen-presenting cells and triggering effective immune stimulatory responses^13^. Upon receiving these signals, activated CD8^+^ T lymphocytes secrete granzyme B (GZMB) and interferon-γ (IFN-γ) to kill tumor cells^14^. By enhancing the immunogenicity of dying tumor cells, necroptosis can potentially synergize with immunotherapies, such as immune checkpoint inhibitors, to improve the clinical outcomes of cancer treatment^10^, ^15^, ^16^. Therefore, the combination of agents that induce necroptosis with immunotherapy presents a potential approach for developing a new generation of cancer therapeutics. CL-387785, a third-generation EGFR inhibitor, has been shown to effectively inhibit the growth of various tumors, including lung cancer, adenomatous polyposis and gastric cancer^17^, ^18^. However, its effect on the tumor immune microenvironment and the specific mechanisms underlying its antimelanoma activity have not yet been reported.

Tumor necrosis factor receptor 1-associated death domain protein (TRADD) is an adaptor protein that plays a significant role in necroptosis^19^, ^20^. TRADD comprises an N-terminal domain that binds to TRAF2 and a C-terminal death domain (DD). Upon recruitment, it forms a complex with the RIPK1, TRAF2, and cIAP proteins^21^. This complex traditionally activates the NF-κB and JNK signaling pathways, resulting in antiapoptotic and proinflammatory responses^22^. It has also been demonstrated that TRADD can induce cell death via necroptosis, where TRADD not only activates RIPK1 to initiate necroptosis but also interacts independently with RIPK3 to determine cell fate^23^. Notably, NF-κB activation in the TRADD signaling pathway plays a vital role in necroptosis-induced immune responses^19^. However, the underlying mechanism through which NF-κB signaling enhances the immune response during necroptosis remains unclear.

In this study, the small-molecule inhibitor CL-387785 exhibited remarkable antitumor activity against melanoma and lung cancer cells via necroptosis. Moreover, CL-387785 enhanced immune-mediated killing of tumor cells through stimulation of the TRADD/RIPK1/NF-κB/CD80 signaling pathway and potentiated the therapeutic efficacy of anti-PD-1 antibodies by upregulating CD80 expression on the tumor cell surface and activating infiltrating CD8^+^ T cells, suggesting that CL-387785 combined with anti-PD-1 therapy represents a potentially viable strategy for cancer treatment.

## 2. Materials and Methods

### 2.1. Cell viability

All cells were purchased from the ATCC. SK-MEL-5, SK-MEL-28, A375 and H1299 cells were cultured in Dulbecco's modified Eagle's medium (DMEM; 11995065; Gibco, China). B16-F10, A549 and PIG1 cell lines were cultured in Roswell Park Memorial Institute (RPMI)-1640 medium (11875093; Gibco) supplemented with 10% fetal bovine serum (FBS; 12J262; ExCell Bio) in an incubator containing 5% CO_2_ at 37 °C. SK-MEL-5, SK-MEL-28, A375, B16-F10, A549, H1299 and PIG1 cells (3×10^3^ cells per well) were inoculated into a 96-well plate, cultured with complete medium supplemented with different concentrations of CL-387785 (HY-10325; MedChemExpress, USA), and then incubated at 37 °C in a thermostatic incubator containing 5% CO_2_ for 24, 48, or 72 hours. Cell viability was determined by measuring the OD value at a wavelength of 450 nm after incubation with a Cell Counting Kit 8 (CCK-8) solution (B34302; Selleck, USA). Each experiment was conducted independently at least three times. The half inhibitory concentration (IC_50_) value was calculated by GraphPad Prism 9.0.

### 2.2. Colony formation assays

SK-MEL-5, SK-MEL-28, A375, and B16-F10 cells (1000 cells per well) were inoculated into a 6-well plate, with three wells in each group. After cell adhesion occurred, the cells were treated with complete culture media supplemented with different concentrations of CL-387785. After 48 hours of cultivation, the supernatant was replaced with complete culture media without CL-387785 for continued cultivation. When visible colonies appeared at the bottom of the plate (approximately two weeks), the colonies were fixed with 4% paraformaldehyde, stained with 0.5% crystal violet (Beyotime, China) and counted by ImageJ software.

### 2.3. Wound healing assay

SK-MEL-5, SK-MEL-28, A375, and B16-F10 cells (1x10^6^ cells per well) were inoculated into a 6-well plate. After the cells adhered to the bottom of the dish, we used a 200 μL pipette tip to create scratches and gently rinsed off the floating cells generated by the scratch with PBS. The cells were then cultured in 2% FBS medium supplemented with different concentrations of CL-387785. After 0 hours of imaging, photos were taken at the same location every 24 hours. The cell healing rate ((0 h-cell wound area - corresponding time point wound area)/0 h-cell wound area) and the wound area size were calculated by ImageJ software.

### 2.4. Transwell assay

We used Transwell cell culture inserts with 8 μM pores (353097; Corning, USA) and 24-well plates in this assay. Matrigel matrix (354248; Corning, USA) was diluted in serum-free DMEM at a ratio of 1:7 on ice, and 60 μL of the mixture was added to each insert and incubated overnight at 37 °C to solidify. The next day, we obtained and resuspended the cells at a density of 5×10^5^/mL in serum-free culture medium supplemented with different concentrations of CL-387785, after which 200 μL was gently added to each insert. At the same time, 500 μL of culture medium supplemented with 30% FBS was added to the lower compartment. After 24 or 48 hours, we wiped off the culture medium and cells that had not passed through the matrix gel in the upper compartment and fixed the cells with 500 μL of 4% paraformaldehyde at room temperature for 15 minutes. Cells on the other side of the membrane were stained with crystal violet for 15 minutes and counted with ImageJ software. Three fields of view for each insert were photographed with an inverted microscope system (Ti-S, Nikon, Tokyo, Japan).

### 2.5. Cell cycle assay

SK-MEL-5, SK-MEL-28, A375, and B16-F10 cells that were pretreated with different concentrations of CL-387785 for 48 hours were collected and fixed overnight at 4 °C with precooled 70% ethanol and kept at -20 °C for at least 2 hours. Afterward, the cells were stained with propidium iodide (PI)/RNase staining solution (C1052; Beyotime, China) and incubated at room temperature in the dark for 15 minutes. Cell detection was performed using flow cytometry (Becton, Dickinson Company, USA), and data analysis was performed using FlowJo software. All the samples were tested three times.

### 2.6. ROS detection

Cells were pretreated with different concentrations of CL-387785 for 6 hours and then digested and resuspended in basal medium to prepare single-cell suspensions. The DCFH-DA reagent (ID3130; Solarbio, China) was diluted 1:1000 and incubated with the cells at 37 °C in the dark for 20 minutes. After incubation, intracellular ROS levels were detected using flow cytometry (Becton, Dickinson Company, USA).

### 2.7. Western blot analysis

Cells were lysed in RIPA lysis buffer containing protease inhibitors and phosphatase inhibitors (B14001; B15001; Selleck, USA), and the protein concentration was detected using the BCA protein quantification kit (20201ES76; Yeasen, China). The protein sample was prepared to a uniform concentration, was added with the corresponding volume of 5× SDS loading buffer (WB3002; NCM Biotech, China), and was denatured in the metal bath at 95 ℃ for 10 minutes. After that, the protein was separated by SDS-PAGE and transferred to a polyvinylidene fluoride membrane (IPVH00010; Millipore, USA). It was sealed with 5% skim milk for 1 hour and incubated overnight with primary antibodies at 4 ℃. On the second day, the membrane was incubated with secondary antibodies at room temperature for 1 hour and imaged using a highly sensitive ECL chemiluminescence assay kit (P10300; NCM Biotech) with chemiluminescence. The primary antibodies involved in our experiments are as listed below: p-MLKL (YM8687; Immunoway, USA), MLKL (A21894; ABclonal, China), p-RIPK1 (66854-1-Ig; Proteintech, USA), RIPK1 (17519-1-AP; Proteintech), Actin (66009-1-Ig; Proteintech), p-JNK (4668S; CST, USA), PARP (9542S; CST), BAX (2772S; CST), GSDMD (ab210070; Abcam, USA), GSDME (ab223877; Abcam), LC3 (YM8147; Immunoway, USA), p65 (8242S; CST), P50 (14220-1-AP; Proteintech).

### 2.8. *In vitro* killing assay

Peripheral blood mononuclear cells (PBMCs) were isolated from the blood samples of healthy volunteers, while murine PBMCs and CD8^+^ T cells were obtained from mouse spleens. Tumor cells were pretreated with or without drugs for 16 hours and then cocultured with isolated immune cells for 24 hours at different effector-to-target cell ratios. The cytotoxicity of the immune cells was then evaluated by measuring the number of live tumor cells after treatment using the CCK-8 assay. Kill ratio (%) = (1-target group/untreated control group) * 100. Approval for the human subjects and animal experimentation was obtained from the Ethics Committee of Xiangya Hospital, Central South University (202308636).

### 2.9. Xenograft tumor model

B16-F10 cells were resuspended in serum-free DMEM, and 5×10^5^ cells were subcutaneously injected into the right backs and abdomens of the mice. When the tumor size reached 50 mm^3^, 24 mice were randomly divided into 4 groups and treated with vehicle + IgG2a, CL-387785 (25 mg/kg) + IgG2a, vehicle + anti-PD-1 (200 μg), or CL-387785 (25 mg/kg) + anti-PD-1 (200 μg) by intraperitoneal injection. The weights of the mice and tumor sizes were monitored daily with a weighing scale and a Vernier caliper. The tumor volumes were calculated using the formula V=0.5×L×W^2^. Approval for the animal experiments was obtained from the Ethics Committee of Xiangya Hospital, Central South University (2023030354).

### 2.10. In vitro pull-down assay

Approximately 1 mg of protein was bound to CL-387785-Sepharose 4B and gently shaken overnight at 4 °C in an incubation buffer solution. Afterward, the beads were washed three times with washing buffer, and the protein of interest was detected by Western blotting.

### 2.11. Quantitative real-time PCR analysis

Total RNA was extracted from B16-F10 cells treated with different concentrations of CL-387785 for different durations with TRIzol reagent. Total RNA (3 μg) served as a template for the reverse transcription reaction (RT Master Mix for qPCR II; HY-K0511A; MedChemExpress, USA). The synthesized cDNA was used as a template for real-time PCR with SYBR Green qPCR mix (B21203; Selleck, USA). All the PCR primers designed and used in this study are listed in Supplementary Table 1.

### 2.12. Ch-IP assay

SK-Mel-5 and SK-Mel-28 cells pretreated with different concentrations of CL-387785 for 24 hours were collected for chromatin immunoprecipitation according to the protocol provided by the EZ ChIP Kit (Millipore, 17-371RF, MA, USA). The soluble lysates were mixed with 5 μL of an anti-p65 antibody and a protease inhibitor and rotated overnight at 4 °C. The primers used to amplify the CD80 promoter region through PCR (50 cycles) are listed in Supplementary Table 2.

### 2.13. Bioinformatics analysis

The data used in this study, including those from the TCGA, Hwang Cohort 2020, and Kim Cohort 2019, were obtained from publicly available databases^24^. These datasets were analyzed using the BEST platform (https://rookieutopia.hiplot.com.cn/), which offers high-resolution analysis of cancer biomarkers through its curated database and innovative analytical pipelines^25^. All analyses were performed following the platform's user manual, which outlines the standard procedures for data processing, normalization, and biomarker exploration.

### 2.14. Statistical analysis

All the data generated in our study are displayed as the mean ± SD, and at least three independent experiments were conducted. All the data were normalized, and significant differences were determined using GraphPad Prism 9.0 with Student's t tests and one-way and two-way ANOVA. p < 0.05 was considered to indicate statistical significance. The number of asterisks indicates the degree of significance (*p < 0.05; **p < 0.01; ***p < 0.001; ****p < 0.0001).

## 3. Results

### 3.1. CL-387785 inhibits the malignant phenotypes in melanoma and lung cancer

**Figure 1:**
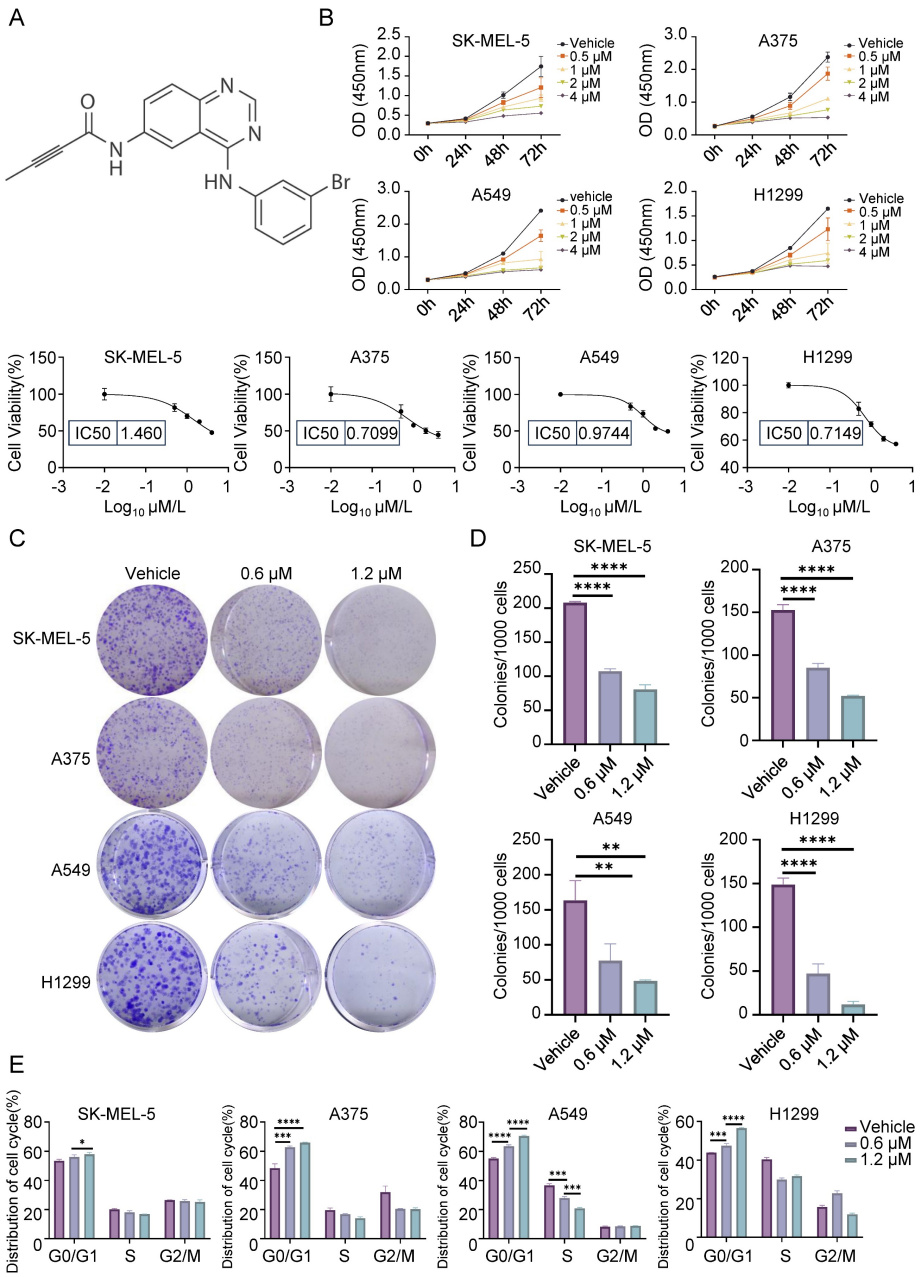
CL-387785 Inhibits the Proliferation of Melanoma and Lung Cancer Cells. (A) Chemical structure of the small-molecule inhibitor CL-387785. (B) CCK-8 assays were conducted to determine the IC_50_ of CL-387785 and its effects on SK-MEL-5, A375 (melanoma), A549, and H1299 (lung cancer) cells. n=4-6. (C) Colony formation assays were performed on melanoma and lung cancer cell lines to evaluate the long-term anti-proliferative effects of CL-387785. n=3. (D) Quantitative analysis of the colony formation assays. (E) The effect of CL-387785 on cell cycle distribution was evaluated via flow cytometry assay. n=3.

To determine the effect of CL-387785 (Fig. 1A) on melanoma and lung cancer cells, various cancer cell lines, including melanoma cell lines and lung cancer cell lines, were treated with CL-387785 at different concentrations. The results indicated that CL-387785 inhibited the proliferation of cancer cells in a time- and concentration-dependent manner, with IC_50_ values of 1.460 μM (SK-MEL-5), 0.7099 μM (A375), 0.9744 μM (A549), 0.7149 μM (H1299), 2.370 µM (SK-MEL-28), and 3.305 μM (B16-F10) (Fig. 1B and Fig. S1A). Furthermore, we evaluated the effects of this drug in additional tumor models, and the results demonstrated that it significantly inhibited HGC-27, SK-OV-3, MDA-MB-231, and U-87 MG cell lines (Fig. S1B). Colony formation assays confirmed the concentration-dependent inhibitory effect of CL-387785 (Fig. 1C, 1D and Fig. S2A). To investigate the mechanism underlying the toxicity of CL-387785, flow cytometry analysis was conducted to assess its effect on the cell cycle, which revealed that CL-387785 induced concentration-dependent G0/G1 phase arrest in both types of cancer cells (Fig. 1E and Fig. S2B).

**Figure 2:**
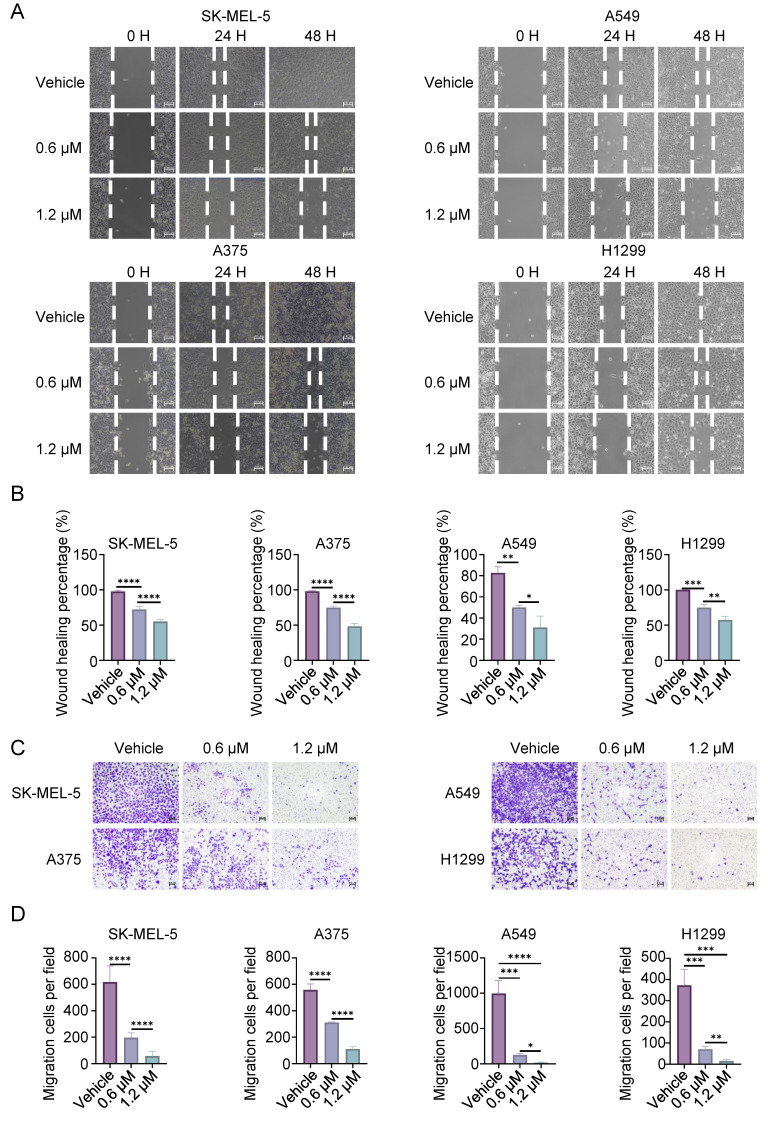
CL-387785 Inhibits Migration and Invasion in Melanoma and Lung Cancer Cells. (A) Wound healing assays were performed to assess the effect of CL-387785 on the migration of SK-MEL-5, A375 (melanoma), A549, and H1299 (lung cancer) cells. ×20 magnification. n=3. (B) Quantitative analysis of the wound healing assays. (C) Transwell assays were conducted to evaluate the inhibitory effect of CL-387785 on invasion. ×20 magnification. n=3. (D) Quantitative analysis of the cell invasion results.

The effects of CL-387785 on the invasion and metastasis of cancer cells were further evaluated. Wound healing assays revealed that CL-387785 inhibited the migration ability of different types of cancer cells (Fig. 2A, 2B, and Fig. S2C). Transwell assays revealed that CL-387785 inhibited the invasive ability of both melanoma and lung cancer cells (Fig. 2C, D, and Fig. S2D). Together, these results suggest that CL-387785 significantly inhibits the proliferation, migration, and invasion of melanoma and lung cancer cells.

### 3.2. CL-387785 induces necroptosis in melanoma and lung cancer cells

**Figure 3:**
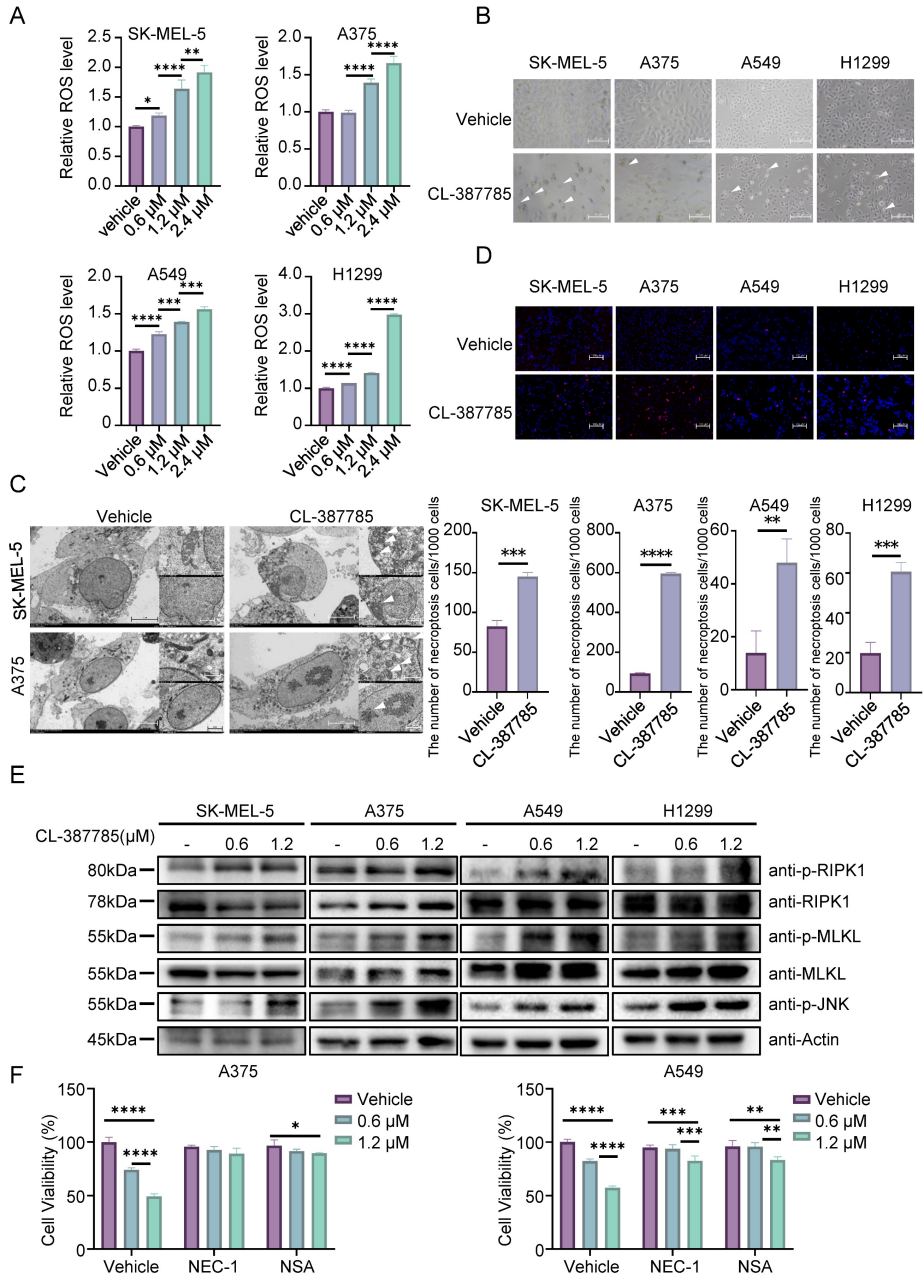
CL-387785 Induces Necroptosis in Tumor Cells. (A) ROS levels in SK-MEL-5, A375 (melanoma), A549, and H1299 (lung cancer) cells were detected after treatment with CL-387785. n=3. (B) The morphological characteristics of SK-MEL-5, A375, A549, and H1299 cells treated with CL-387785 were observed by light microscopy. ×20 magnification. (C) Necroptotic features of melanoma cells were further visualized using transmission electron microscopy after CL-387785 treatment. (D) Hoechst/PI staining was performed to assess necroptotic cell death in melanoma and lung cancer cells treated with CL-387785. Quantitative analysis of the Hoechst/PI staining is shown in the image. ×20 magnification. n=3. (E) Western blot analysis was used to assess the protein levels of phosphorylated RIPK1 (p-RIPK1), RIPK1, phosphorylated MLKL (p-MLKL), MLKL, and phosphorylated JNK (p-JNK) in SK-MEL-5, A375, A549, and H1299 cell lysates. (F) Rescue effects of NEC-1 and NSA on CL-387785-induced cell death in A375 and A549 cells. n=4.

To further investigate the specific form of cell death induced by CL-387785, the expression levels of apoptosis-, autophagy- and pyroptosis-related markers in melanoma and lung cancer cells after drug treatment were detected by Western blotting. Cleavage of PARP, LC3, GSDMD or GSDME was not detected after CL-387785 treatment, and increased expression of the apoptotic protein BAX was not detected (Fig. S3A). Interestingly, ROS levels increased significantly in the cancer cells after treatment (Fig. 3A and Fig. S3B, 3C). Next, the ferroptosis inhibitor Fer-1 was utilized to evaluate whether CL-387785 induces cell death via ferroptosis, which is an intracellular iron-dependent form of lipid peroxide death. Fer-1 treatment did not reduce the degree of cell death caused by CL-387785 (Fig. S3D), excluding the possibility of ferroptosis. These data suggest that CL-387785 does not exert its cytotoxic effects by inducing apoptosis, autophagy, pyroptosis, or ferroptosis.

Typical swelling was observed in the cells after drug intervention (Fig. 3B and Fig. S3E), and morphological changes including membrane rupture, vacuolar-like changes, mitochondrial swelling, mitochondrial cristae disorder, nuclear rupture, and other necrotic morphological changes were observed in melanoma cells treated with CL-387785 using transmission electron microscopy (Fig. 3C). After 48 hours of treatment, the nuclei of the cancer cells in the control group exhibited diffuse and uniform blue fluorescence with a relatively small proportion of cells emitting red fluorescence, whereas the proportion of cells emitting red fluorescence increased significantly in the CL-387785-treated group. (Fig. 3D and Fig. S3F), indicating that CL-387785 treatment induced necroptosis in different types of cancer cells. In addition, necroptosis inhibitors reversed CL-387785-induced cell death (Fig. S3G). Immunoblotting was conducted to detect the expression of key molecules involved in the intracellular necroptosis pathway after treatment with different concentrations of CL-387785. CL-387785 treatment significantly increased the expression levels of p-RIPK1 and p-MLKL (Fig. 3E and Fig. S4A). Moreover, necrostatin-1 (Nec-1) and necrosulfonamide (NSA) effectively suppressed the antitumor activity of CL-387785 (Fig. 3F). Since CL-387785 is an EGFR inhibitor, we investigated whether EGFR contributes to necroptosis induction. EGFR knockdown did not upregulate the expression of necroptosis-associated proteins (Fig. S4B). Together, these results suggest that CL-387785 induces necroptosis in melanoma and lung cancer cells in an EGFR-independent manner.

### 3.3. CL-387785 sensitizes tumors to anti-PD-1 mAb therapy through the activation of tumor-infiltrating CD8^+^ T cells

**Figure 4:**
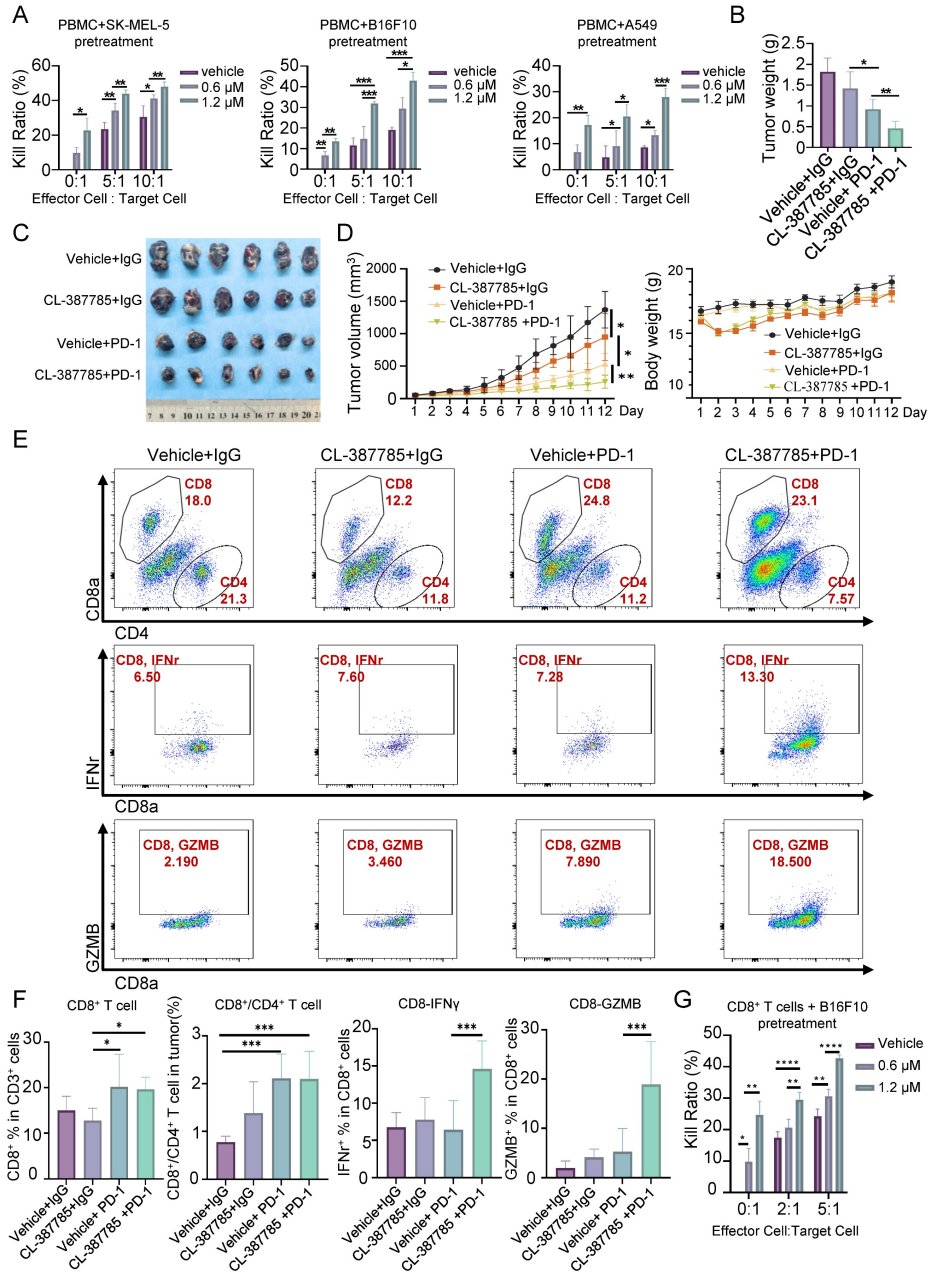
CL-387785 Enhances the Efficacy of Anti-PD-1 Therapy by Modulating CD8^+^ T Cells. (A) An in vitro cytotoxicity assay was performed to assess the ability of CL-387785 to enhance the cytotoxic function of PBMCs. n=4. (B-D) B16-F10 tumor-bearing C57BL/6 mice were treated with CL-387785, IgG2a, or an anti-PD-1 mAb, and the effects on tumor weight, tumor volume, and body weight were evaluated. n=6. (E, F) Flow cytometry was used to determine the populations of CD8^+^ T cells, CD8^+^/CD4^+^ T cells, IFN-γ^+^ CD8^+^ T cells, and GZMB^+^ CD8^+^ T cells in B16-F10 tumors. n=6. (G) An in vitro cytotoxicity assay was performed to assess the ability of CL-387785 to enhance the cytotoxic function of CD8^+^ T cells. n=4.

Necroptosis is a type of immunogenic death that can activate adaptive immune responses and produce long-lasting antitumor immune responses. We then assessed the effect of CL-387785 on the immune microenvironment in treated tumors by pretreating tumor cells with CL-387785 and then coculturing them with PBMCs. The results indicated that CL-387785 pretreatment effectively increased the killing capacity of immune cells in vitro, which was subsequently abrogated by necrostatin-1 (Fig. 4A and Fig. S4C). Given that combination therapy is an effective way to promote immunotherapy efficacy, we further explored whether CL-387785 could enhance the therapeutic efficacy of anti-PD-1 mAbs in a melanoma mouse model (Fig. 4B-E). C57BL/6 mice bearing B16-F10 tumors were treated with CL-387785, an anti-PD-1 mAb, CL-387785 plus an anti-PD-1 mAb, or an IgG isotype control (IgG2a). Compared with mice treated with IgG2a, mice treated with CL-387785 and the anti-PD-1 mAb demonstrated attenuated tumor growth. Moreover, compared with those in mice treated with CL-387785 or the anti-PD-1 mAb alone, tumor growth in the combination treatment group was notably inhibited, with minimal effects on body weight and organs (Fig. 4B-4D and Fig. S4D, 4E).

To explore the downstream mechanism through which CL-387785 contributes to anti-PD-1 therapy sensitivity, the percentages of tumor-infiltrating CD8^+^ T cells, DCs, Tregs and MDSCs were analyzed. Compared with those in the groups treated with CL-387785 alone and IgG2a, the percentages of CD8^+^/CD4^+^ T cells in the groups treated with the anti-PD-1 mAb as well as CL-387785 combined with the anti-PD-1 were significantly greater, while the numbers of DCs, Tregs, NK cells and MDSC cells did not significantly differ, indicating that the efficacy of both groups was closely related to the number of CD8^+^ T cells rather than the number of DCs and Tregs (Fig. 4E, 4F, and Fig. S5A-D). Notably, although the combination treatment group showed greater therapeutic efficacy than the anti-PD-1 mAb group did, the number of infiltrating CD8^+^ T cells did not significantly differ between these groups, suggesting that the difference in efficacy is not due to the number of tumor-infiltrating CD8^+^ T cells but to their function. Perhaps CD8^+^ T cells in the tumor microenvironment (TME) were functionally incompetent and could be activated by CL-387785 treatment in the combination treatment group. Thus, the activity of the infiltrating CD8^+^ T cells was tested in both groups. Consistent with our hypothesis, the activity of CD8^+^ IFN-γ^+^ T cells and CD8^+^GZMB^+^ T cells significantly increased in the combined treatment group, indicating an increase in the cytotoxicity of infiltrating CD8^+^ T cells. To determine whether the cytotoxic T lymphocytes originated from the periphery, we analyzed CD8^+^ T lymphocytes in the spleen. The results revealed that the function of CD8^+^ T lymphocytes in the system was not significantly affected by CL-387785 treatment (Fig. S6A). Together, these results demonstrated that CL-387785 could improve the therapeutic efficacy of anti-PD-1 mAbs by increasing the cytotoxicity of tumor-infiltrating CD8^+^ T cells.

### 3.4. CL-387785 activates tumor-infiltrating CD8^+^ T cells by increasing CD80 protein levels on the cancer cell surface

The classical immune process in tumors includes the identification and processing of tumor cells by antigen-presenting cells, the recognition of processed tumor cells by CD8^+^ T cells, and the eventual activation of CD8^+^ T cells to kill the tumor cells. In previous animal experiments, the cytotoxicity of tumor-infiltrating CD8^+^ T cells was significantly increased by CL-387785 in the combination treatment group, whereas the cytotoxicity of DCs, the major antigen-presenting cells in tumors, was not increased by CL-387785. Therefore, we speculate that the activation of CD8^+^ T cells induced by CL-387785 treatment is mediated by direct regulation of cancer cells. To test this hypothesis, B16-F10 melanoma cells were pretreated with CL-387785 for 16 hours and then cocultured with CD8^+^ T lymphocytes isolated from mouse spleens. The results showed that CL-387785 pretreatment significantly improved the ability of CD8^+^ T cells to kill tumor cells in the absence of antigen-presenting cells, confirming our hypothesis (Fig. 4G). Interestingly, pharmacological blockade of TLR4, a key DAMP receptor, did not completely attenuate the cytotoxic activity of CD8^+^ T lymphocytes, suggesting the involvement of TLR4 in the CL-387785-induced activation of CD8^+^ T lymphocytes (Fig. S6B).

**Figure 5:**
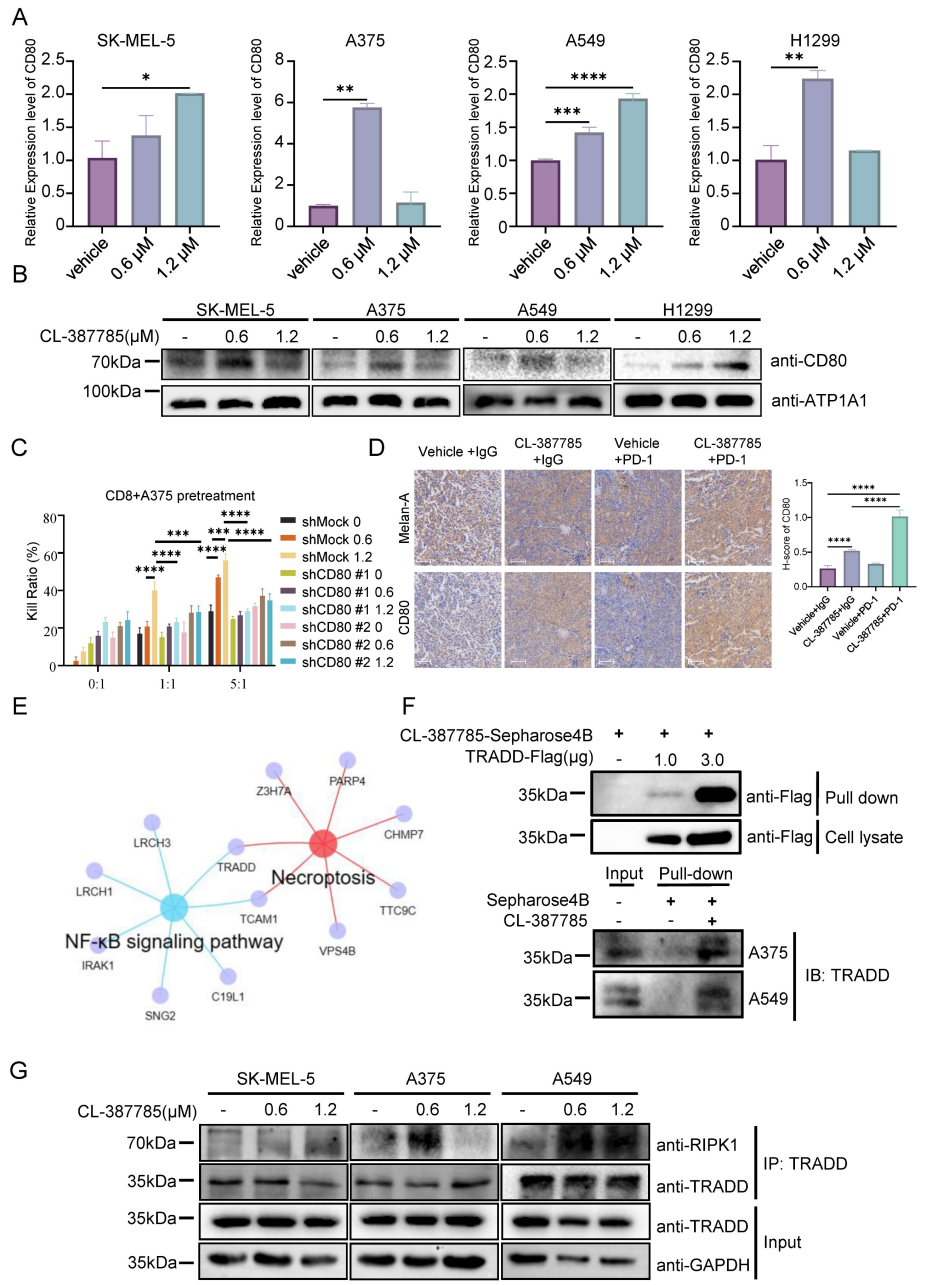
CL-387785 targets TRADD to regulate CD80 expression on the surface of tumor cells. (A) RT‒PCR was used to analyze the expression levels of CD80 in SK-MEL-5, A375, A549, and H-1299 cells after treatment with CL-387785. n=3. (B) Membrane proteins were extracted from SK-MEL-5, A375, A549, and H-1299 cells treated with CL-387785 to evaluate CD80 protein expression. (C) In vitro cytotoxicity assays were performed to assess the effect of CD80 knockdown on the immunostimulatory effects of CL-387785 on CD8^+^ T cells. n=6. (D) Immunohistochemical staining was used to detect CD80 and Melan-A (YM8267; Immunoway) expression in B16-F10 tumors. n=6. ×20 magnification. (E) A Venn diagram illustrating genes shared between necroptotic and NF-κB signaling pathways identified by IP-MS. (F) Endogenous and exogenous PULL-DOWN assays were conducted in melanoma samples using CL-387785, and TRADD expression was detected via Western blotting. (G) Immunoprecipitation experiments with TRADD in SK-MEL-5, A375, and A549 cell lysates were performed, followed by Western blotting to detect RIPK1 and TRADD protein expression.

Tumor cells can activate T cells through antigen signals or costimulatory signals, among which antigen signals are related to the specific characteristics of the immunogen. With respect to melanoma, the most common antigen signals are melanoma differentiation antigens and major histocompatibility complex (MHC) molecules, and costimulatory signals refer to immune costimulatory molecules such as CD80/86. To explore the mechanism by which CL-387785 contributes to the regulation of tumor cells, we measured the expression of these signals in CL-387785-pretreated melanoma cells by real-time PCR. The results revealed that the expression of the costimulatory molecule CD80 significantly increased, whereas the expression of melanoma differentiation antigens and MHC did not significantly change (Fig. 5A and Fig. S6C). Furthermore, CD80 expression on the B16-F10 melanoma cell surface increased significantly in a concentration-dependent manner after the cells were incubated with CL-387785 for 24 hours (Fig. 5B and Fig. S6D). We conducted in vitro killing experiments by knocking down CD80 expression in B16-F10 cells using shRNA and reported that the killing effect of CD8^+^ T cells on B16-F10 cells with CD80 knockdown was significantly reduced (Fig. 5C and Fig. S6E). To confirm these findings in vivo, we conducted immunohistochemistry on tumor tissues obtained from C57BL/6 mice bearing B16-F10 tumors subjected to different treatments, including CL-387785, anti-PD-1 mAb, CL-387785 plus anti-PD-1 mAb, and IgG isotype control treatments. In line with the in vitro results, mice treated with CL-387785 presented increased expression of CD80, especially in the combination treatment group (Fig. 5D). Together, these data demonstrate that CL-387785 activates tumor-infiltrating CD8^+^ T cells by increasing CD80 protein levels on the cancer cell surface to increase the efficacy of anti-PD-1 mAb therapy.

Since CL-387785 has been reported to be an EGFR inhibitor, to investigate the specific mechanism through which CL-387785 induces CD80 expression, we investigated the relationship between EGFR expression and CD80 expression. Both public data and RT‒PCR results indicated that EGFR does not significantly promote CD80 expression (Fig. S7A, 7B). Proteomic and bulk RNA-seq results suggested that CL-387785 intervention affected TRADD and TCAM1 expression in tumor cells (Fig. 5E). The results of surface plasmon resonance, as well as endogenous and exogenous pull-down experiments, further validated the high-throughput sequencing results (Fig. 5F and Fig. S7C), indicating that CL-387785 could recruit TRADD within cells. Additionally, the results of endogenous immunoprecipitation demonstrated that CL-387785 promoted the binding of TRADD and RIPK1, suggesting that TRADD may function through RIPK1 (Fig. 5G and Fig. S7D). Moreover, in vitro cytotoxicity experiments revealed that knockdown of TRADD and RIPK1 expression significantly inhibited the cytotoxicity of immune cells to tumor cells (Fig. S7E).

### 3.5. CL-387785 increases tumor immunotherapy efficacy by regulating CD80 expression via the modulation of NF-κB nuclear translocation

**Figure 6:**
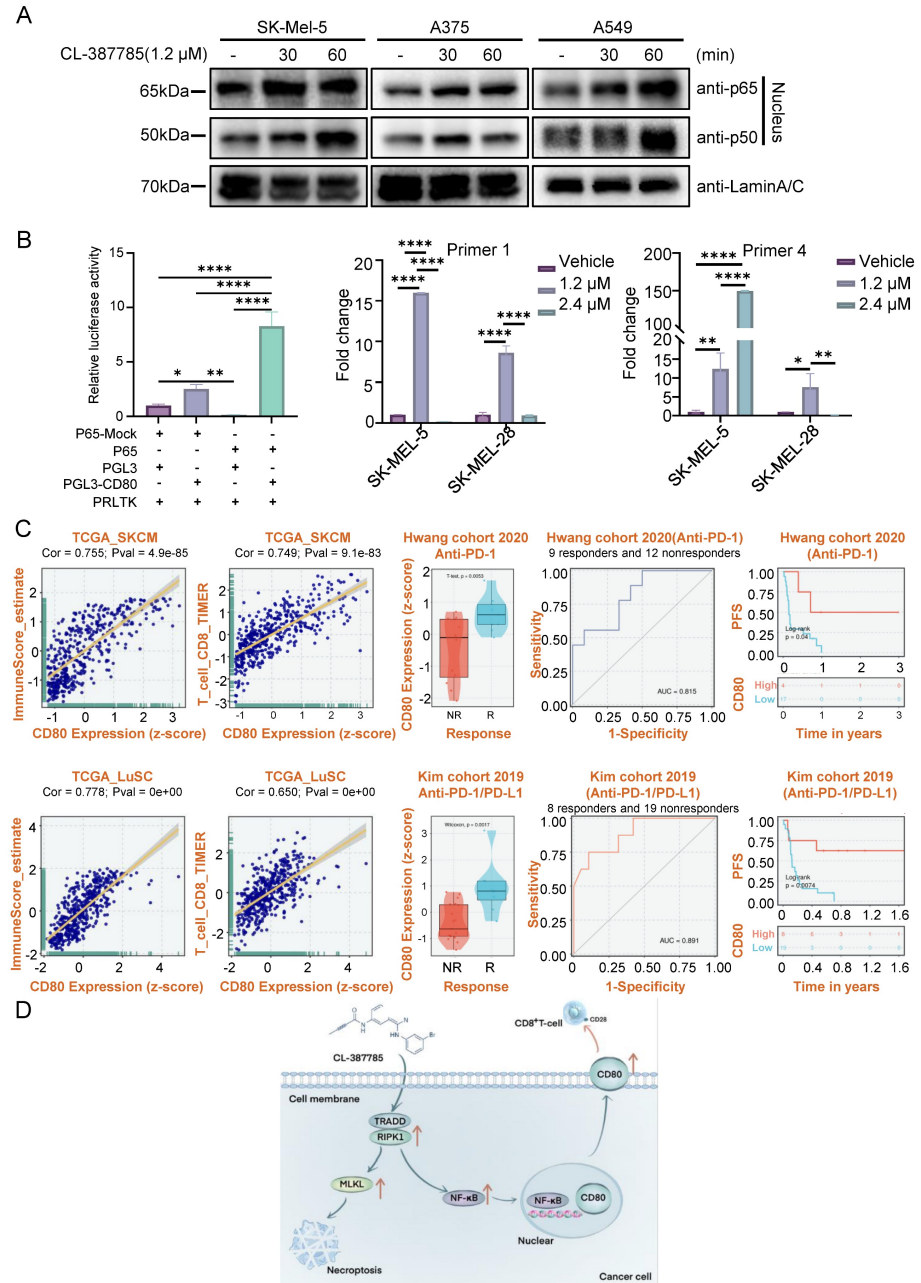
CL-387785 affects CD80 transcription by regulating the nuclear translocation of NF-κB. (A) Western blotting was used to detect the protein expression of P65 and P50 in SK-MEL-5, A375 and A549 cells. (B) Chip-PCR (n=3) and luciferase reporter gene assays (n=4) were used to examine the mechanism underlying the transcriptional regulation of CD80 by P65. (C) The clinical significance of CD80 expression in the melanoma and lung cancer cohorts.

Studies have shown that NF-κB signaling in necroptotic cells is necessary for the induction of a CD8^+^ T-cell response and that NF-κB activation can increase the expression of CD80; however, the underlying mechanism remains unclear. We treated SK-MEL-5, A375 and A549 cells with 1.2 μM CL-387785 for different durations, after which nuclear and plasma proteins were extracted to detect the activation state of NF-κB. The results revealed that as the intervention time increased, the levels of P65 and P50 in the nucleus increased, and NF-κB activity increased (Fig. 6A). Moreover, genetic or pharmacological inhibition of TRADD, RIPK1, or NF-κB abolished the ability of CL-387785 to upregulate CD80 expression and activate the NF-κB signaling pathway (Fig. S8A). Therefore, we examined p65, the putative target of CD80, by a luciferase reporter assay. Compared with the control group, p65 increased luciferase activity nearly 60-fold through binding to a target sequence (Fig. 6B). Furthermore, chromatin immunoprecipitation (ChIP)-PCR was conducted to determine the exact binding area of P65. The results indicated that CL-387785 significantly increased the binding of P65 to CD80 Primer 1 (-722 to -955 bp) and Primer 2 (-1712 to -1870 bp) (Fig. 6B and Fig. S8B).

Transcriptomic analysis of data from The Cancer Genome Atlas (TCGA) revealed a positive correlation between CD80 expression and immune score in melanoma and lung cancer tumors, particularly the immune score of CD8-positive T lymphocytes. These findings suggest that increased CD80 expression is linked to increased immune cell infiltration in the tumor microenvironment. Remarkably, among patients receiving anti-PD-1 therapy, the level of CD80 expression is elevated in responders, indicating that it is an effective predictor of the response to anti-PD-1 treatment (Fig. 6C). Furthermore, patients with high CD80 expression have prolonged progression-free survival. To summarize, these findings highlight the significance of upregulating CD80 expression as a critical strategy to increase the efficacy of immunotherapy.

## 4. Discussion

With the use of immune checkpoint blockade (ICB), tremendous progress has been made in treating cancers such as melanoma and lung cancer^26^, ^27^. Nevertheless, only a subset of patients exhibit robust responses to ICB, and resistance to ICB remains a clinical challenge, which is possibly attributed to insufficient TIL infiltration and low immune cell activity^28^. Altering the tumor immune microenvironment (TME) can effectively promote the therapeutic efficacy of ICB^29^, ^30^. Programmed cell death (PCD) plays vital roles in regulating the immunosuppressive tumor microenvironment, thus determining the outcomes of clinical tumor therapies^10^, ^31^. The most intensively studied types of PCD are apoptosis, pyroptosis, necroptosis, PANoptosis, ferroptosis, and autophagy, which can be further categorized into immunogenic and nonimmunogenic (or tolerogenic) processes on the basis of the ability to initiate further adaptive immune response. In our study, we confirmed that CL-387785 inhibited melanoma and lung cancer growth by inducing necroptosis. Necroptosis, a type of immunogenic PCD, can warn the surrounding immune system of potential dangers via the release of cellular components, such as proinflammatory cytokines or other DAMPs^10^, ^15^, ^32^, which can be further identified by pattern recognition receptors on innate immune cells, ultimately enhancing the efficacy of immunotherapy. In recent years, several protocols leveraging necroptosis to enhance the efficacy of immune checkpoint therapies have been proposed, including in situ vaccine therapy, photodynamic technology, and cryoablation, which aim to reduce immune tolerance in malignant tumors such as breast and lung cancers^15^, ^33^-^35^. In our study, CL-387785 pretreatment effectively increased the capacity of immune cells to kill tumor cells in vitro and enhanced the therapeutic efficacy of anti-PD-1 mAbs in a melanoma mouse model. Given that inducing necroptosis in cancer cells has become a novel strategy for enhancing the efficacy of tumor immunotherapy, CL-387785 in combination with anti-PD-1 mAbs is a potentially combination therapy for melanoma and lung cancer.

The antitumor immune response induced by necroptotic cells is reportedly associated with the cross-activation and proliferation of CD8^+^ T cells^10^, ^36^. In hepatocellular carcinoma, key necroptosis-associated genes, such as RIPK1, RIPK3, and p-MLKL, are positively correlated with CD3^+^ and CD8^+^ T-cell densities in the TME, suggesting a better prognosis for patients^37^. In breast cancer, the intratumoral injection of necroptotic tumor cells increases the recruitment of CD8^+^ T cells into the TME, enhancing the efficacy of ICB^38^. In addition to inducing potent antitumor immunity by increasing the number of CD8^+^ T cells, necroptotic cells can increase the cytotoxicity of CD8^+^ T cells by regulating IFN-γ secretion^39^, ^40^. Additionally, dying cells release DAMPs, ATP, and HMGB1, which can deliver antigenic and inflammatory stimuli to dendritic cells to initiate adaptive immunity, eventually activating CD8^+^ T cells through antigen cross-priming^36^, ^41^. In the exploration of the downstream mechanism through which CL-387785 contributes to anti-PD-1 therapy sensitivity, no significant difference in the number of infiltrating CD8^+^ T cells was detected after CL-387785 treatment, suggesting that the difference in efficacy is not due to the number but rather to the function of infiltrating CD8^+^ T cells. Further evaluations of tumor-infiltrating CD8^+^ T-cell activity confirmed the increase in cytotoxicity, as evidenced by the increase in the activity of CD8^+^ IFN-γ^+^ T cells and CD8^+^GZMB^+^ T cells. Thus, CL-387785 could increase the therapeutic efficacy of anti-PD-1 mAbs by increasing the cytotoxicity of tumor-infiltrating CD8^+^ T cells, providing a novel drug treatment for ICB-resistant patients.

The classical antitumor immune response emphasizes the iterative nature of the response, in which antigen-presenting cells present MHC class I molecules, tumor-specific antigens, and neoantigens to T lymphocytes, thereby activating CD8^+^ T lymphocytes^42^. In this process, various types of costimulatory molecules provide secondary signals that enhance T-cell activation and ensure an effective immune response. In addition to the classical pathway, when tumor cells highly express costimulatory molecules, they can present antigens directly to CD8^+^ T cells and stimulate their differentiation into cytotoxic T lymphocytes^43^. To further investigate the underlying mechanism of CD8^+^ T-cell activation, we conducted q-PCR and found that CL-387785 increased CD80 expression on the tumor cell surface in a dose-dependent manner. CD80 is an important costimulatory molecule that can stimulate the T-cell immune response by promoting T-cell proliferation, increasing cytokine secretion, and preventing apoptosis^44^. In tumors of various types, such as non-small cell lung cancer, pancreatic cancer, and lymphoma, the overexpression of CD80 on the tumor cell surface contributes to the infiltration and antitumor function of immune cells, while insufficient or decreased expression of CD80 leads to T lymphocyte dysfunction^45^-^49^. In our study, CL-387785 activated CD8^+^ T cells through the overexpression of the costimulatory molecule CD80 on the tumor cell surface.

Although previous studies have shown that necroptotic cells can upregulate CD80 expression on the cell membrane surface, the exact mechanism remains unclear^50^. TRADD, a downstream effector of TNF, typically binds to RIPK1 and TRAF2, recruiting Caspase-8 and FADD and thus inducing apoptosis^23^. In the absence of caspase-8, p-RIPK1 can upregulate p-RIPK3 and p-MLKL, consequently activating the NF-κB signaling pathway and leading to necroptosis^20^. In our experiments, we demonstrated that CL-387785 induces the upregulation of p-RIPK1 and p-MLKL, resulting in necroptosis. It is well known that the TRADD-RIPK1 complex can activate downstream NF-κB and MAPK signaling pathways, and in turn, activated NF-κB promotes necroptosis by inhibiting Caspase-8 activity^51^. The inhibition of TRADD was reported to impair necroptosis by disrupting the phosphorylation of RIPK1 and NF-κB^22^. Findings from Yatim's group suggest that CD8^+^ T-cell cross-priming requires signals from both RIPK1 and the NF-κB signaling pathway, with either NF-κB signaling or necroptosis alone reducing priming efficiency and tumor immunity^36^. Interestingly, we found that CL-387785 targeted TRADD and strengthened its interaction with RIPK1, ultimately activating the NF-κB signaling pathway. We further confirmed that NF-κB binds to the promoter region of CD80, promoting its transcription. Collectively, these results indicate that CL-387785 upregulates CD80 expression on tumor cell surfaces through an NF-κB-dependent mechanism, thereby enhancing CD8^+^ T-cell cytotoxicity.

## 5. Conclusions

In summary, our study reveals that CL-387785 coordinates multiple processes that are critical for both the inhibition of malignant cancer cell phenotypes and the activation of tumor-infiltrating CD8^+^ T cells. CL-387785 effectively suppresses the malignant phenotype of melanoma and lung cancer cells through TRADD/RIPK1-induced necroptosis. In addition, tumor-infiltrating CD8^+^ T cells could be activated by CL-387785-induced CD80 overexpression in cancer cells through NF-κB, consequently enhancing the efficacy of anti-PD-1 treatment. Hence, this study provides a proof-of-concept for a promising therapeutic approach to combine immunogenic cell death with immunotherapy in cancer therapy.

## Supplementary Material

Supplementary figures and tables.

## Abbreviations

CCK-8: Cell Counting Kit-8
CHIP: Chromatin Immunoprecipitation
cIAP: Cellular Inhibitor of Apoptosis
CO-IP: Co-Immunoprecipitation
CTL: Cytotoxic T Lymphocyte
CTLA-4: Cytotoxic T Lymphocyte-associated Antigen-4
DAMP: Damage Associated Molecular Patterns
DC: Dendritic Cell
DD: Death Domain
EGFR: Epidermal Growth Factor Receptor
GZMB: Granzyme B
IC_50_: Half maximal inhibitory concentration
ICB: Immune Checkpoint Blockade
ICD: Immunogenic Cell Death
IFN-γ: Interferon-γ
JNK: c-Jun N-terminal Kinase
mAbs: Monoclonal antibodies
MDSC: Myeloid-Derived Suppressor Cell
MHC: Major Histocompatibility Complex
MLKL: Mixed lineage kinase domain-like
NF-κB: Nuclear factor κB
NK: Natural Killer Cell
PBMC: Peripheral Blood Mononuclear Cells
PCD: Programmed Cell Death
PD-1: Programmed Death 1
qRT-PCR: Quantitative Reverse Transcription Polymerase Chain Reaction
RIPK1: Receptor interacting serine/threonine kinase 1
RIPK3: Receptor interacting serine/threonine kinase 3
ROS: Reactive Oxygen Species
TCGA: The Cancer Genome Atlas
TME: Tumor Microenvironment
TRADD: Tumor Necrosis Factor Receptor 1-Associated Death Domain Protein
TRAF2: TNF receptor associated factor 2
Treg: Regulatory T Cell
WB: Western Blot
