# Supplementary material for: Targeted Induction of Cancer Cell Necroptosis Potentiates Anti-PD-1 Immunotherapy via CD80 Activation
Source: Int J Biol Sci. 2026 Jan 22;22(4):2085–100. doi: 10.7150/ijbs.121690 (PMC12905644; doi:10.7150/ijbs.121690)
Supplement: Supplementary file 1 — Supplementary figures and tables. [file ijbsv22p2085s1.pdf]

Supplementary materials

Targeted Induction of Cancer Cell Necroptosis Potentiates Anti-PD-1 Immunotherapy via CD80 Activation

Xu Zhang[1,2,3,4,5†], Detian Zhang[1,2,3,4,5,6†], Zhe Zhou[1,2,3,4,5], Waner Liu[1,2,3,4,5], Susi Zhu[1,2,3,4,5], Siyu Xiong[1,2,3,4,5], Xiang Chen[1,2,3,4,5,6*], Cong Peng[1,2,3,4,5*]

[1]The Department of Dermatology, Xiangya Hospital, Central South University, Changsha, Hunan 410008, China. [2]Furong Labratory, Changsha, Hunan 410028, China. [3]Hunan Key Laboratory of Skin Cancer and Psoriasis, Xiangya Hospital, Central South University, Changsha, Hunan 410008, China. [4]Hunan Engineering Research Center of Skin Health and Disease, Xiangya Hospital, Central South University, Changsha, Hunan 410008, China. [5]National Clinical Research Center for Geriatric Disorders, Xiangya Hospital, Central South University, Changsha, Hunan 410008, China. [6]Key Laboratory of Traditional Chinese Medicine Syndrome / National Institute of Extremely-Weak Magnetic Field Infrastructure, Hangzhou 310028, China.

*Address correspondence to: pengcongxy@csu.edu.cn (C.P.) and chenxiangck@126.com (X.C.)

†X.Z. and D.Z. contributed equally to this work.

This Word file includes:

Figs. S1 to S8

Tables S1 to S2

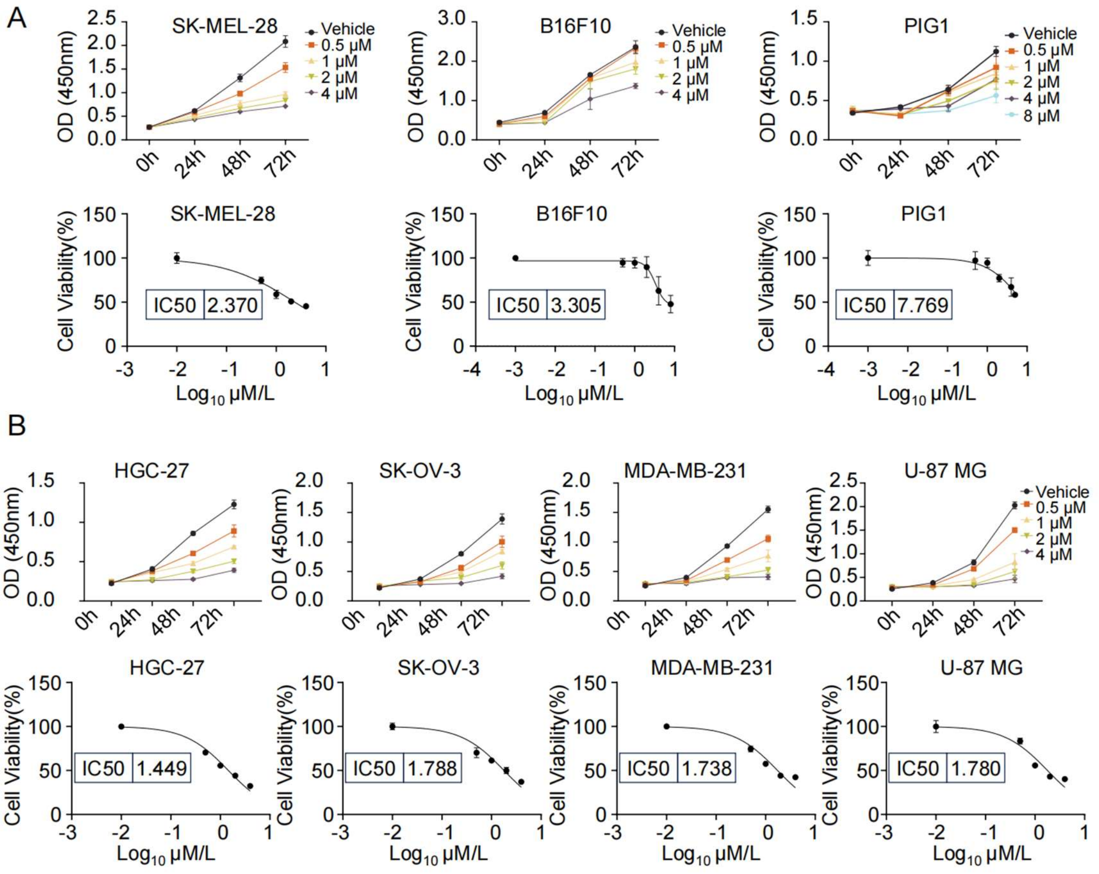

Fig. S1. CL-387785 inhibits the proliferation of multiple tumor cell lines. (A) CCK-8 assays were conducted to determine the IC50 of CL-387785 and its effects on SK-MEL-28, B16F10 and PIG1 cells. n=5. (B) CCK-8 assays were performed to determine the IC50 of CL-387785 and its effects on HGC-27, SK-OV-3, MDA-MB-231, and U-87 MG cells. n=5.

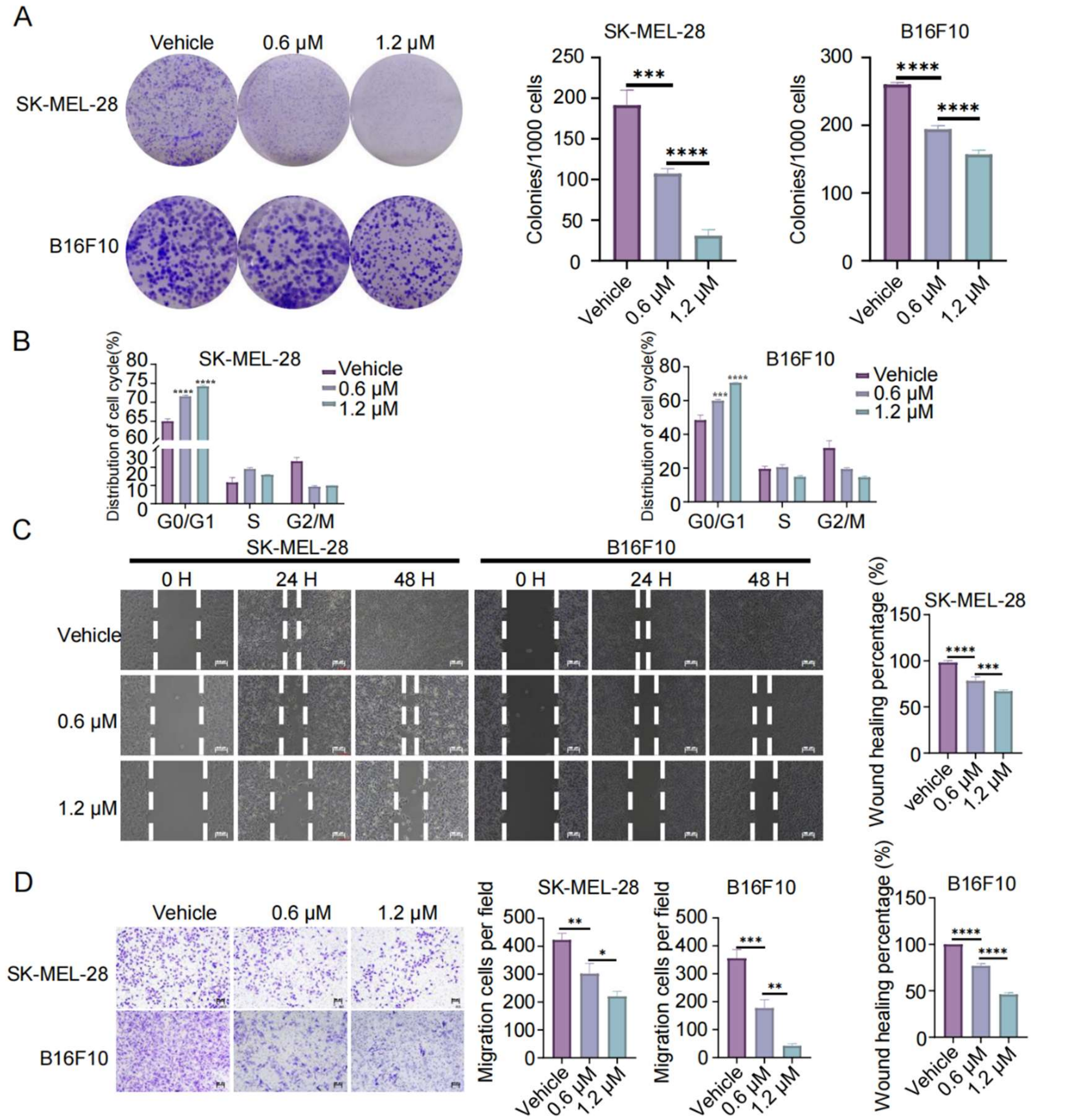

Fig. S2. CL-387785 inhibits the malignant phenotype of melanoma cells. (A) Colony formation assays were performed on SK-MEL-28 and B16F10 cells to evaluate the long-term antiproliferative potential of CL-387785. The results of the quantitative analysis of the colony formation assays are listed in the right panel. n=3. (B) The effect of CL-387785 on cell cycle distribution was evaluated via flow cytometry. n=3. (C) Wound healing assays were performed to assess the effect of CL-387785 on the migration of SK-MEL-28 and B16F10 cells. The results of the quantitative analysis

of the wound healing assays are shown in the right panel. × 20 magnification. n=3. (D) Transwell assays were conducted to evaluate the inhibitory effect of CL-387785 on cell invasion. Quantitative analysis of the cell invasion results is shown in the right panel. ×20 magnification. n=3.

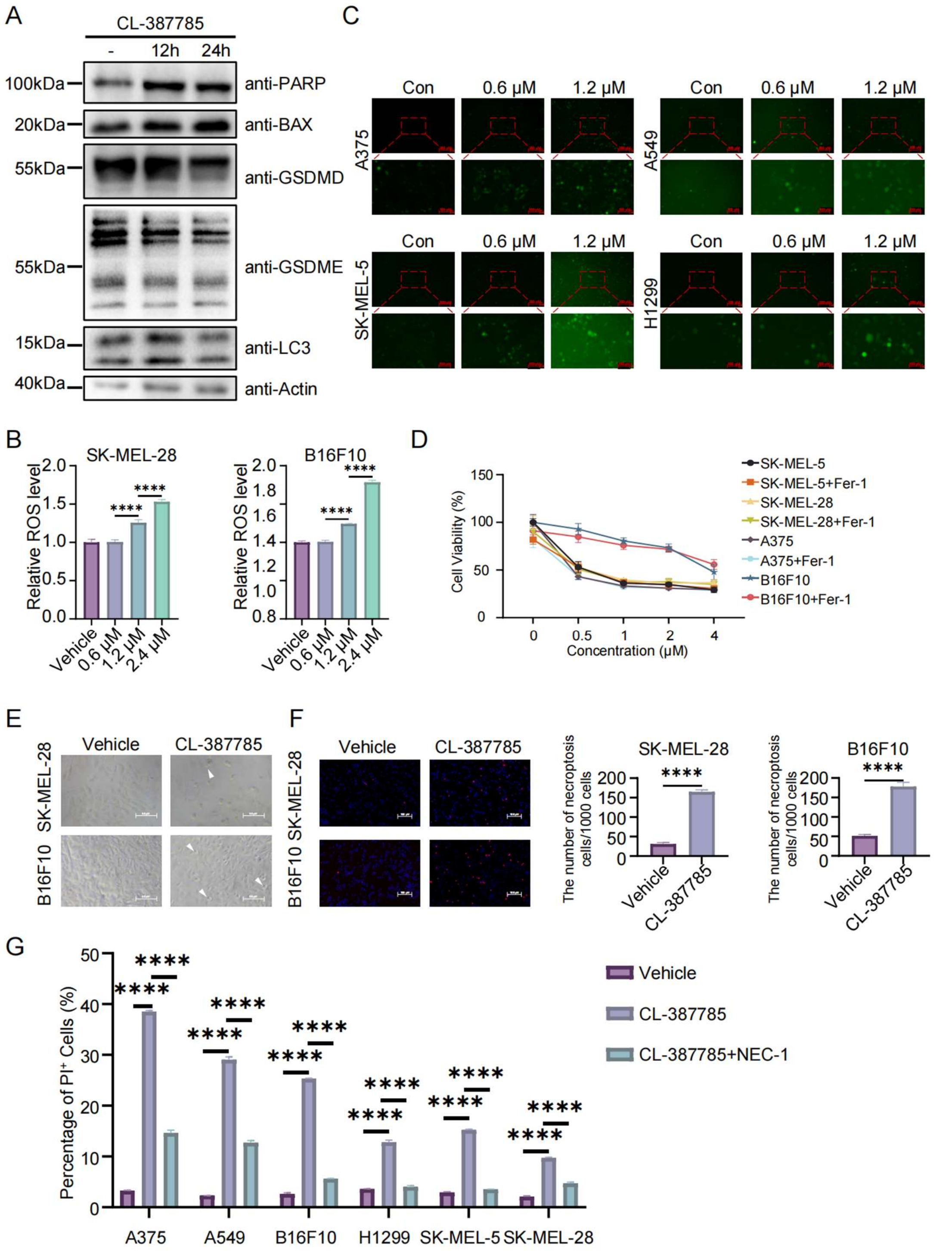

Fig. S3. CL-387785 Induces Necroptosis in Tumor Cells. (A) Western blot analysis was used to assess the protein levels of PARP, BAX, GSDMD, GSDME and LC3 in SK-MEL-5 cell lysates after treatment with CL-387785. (B) DCFH-DA staining was used to assess ROS levels in A375,

A549, SK-MEL-5, and H1299 cells following CL-387785 treatment. (C) ROS levels in SK-MEL-28 and B16F10 cells were detected after treatment with CL-387785. ×20 magnification. n=3. (D) Fer-1 was used to exclude the role of ferroptosis in the effects of CL-387785 intervention. n=6. (E) The morphological characteristics of SK-MEL-28 and B16F10 cells treated with CL-387785 were observed by light microscopy. × 20 magnification. (F) Hoechst/PI staining was performed to assess necroptotic cell death in SK-MEL-28 and B16F10 cells treated with CL-387785. Quantitative analysis of the Hoechst/PI staining is shown in the right panel. ×20 magnification. n=3. (G) Annexin V-PI staining was used to measure necroptosis levels in A375, A549, B16F10, H1299, SK-MEL-5, and SK-MEL-28 cells induced by CL-387785 under necroptosis-inhibiting conditions. n=3.

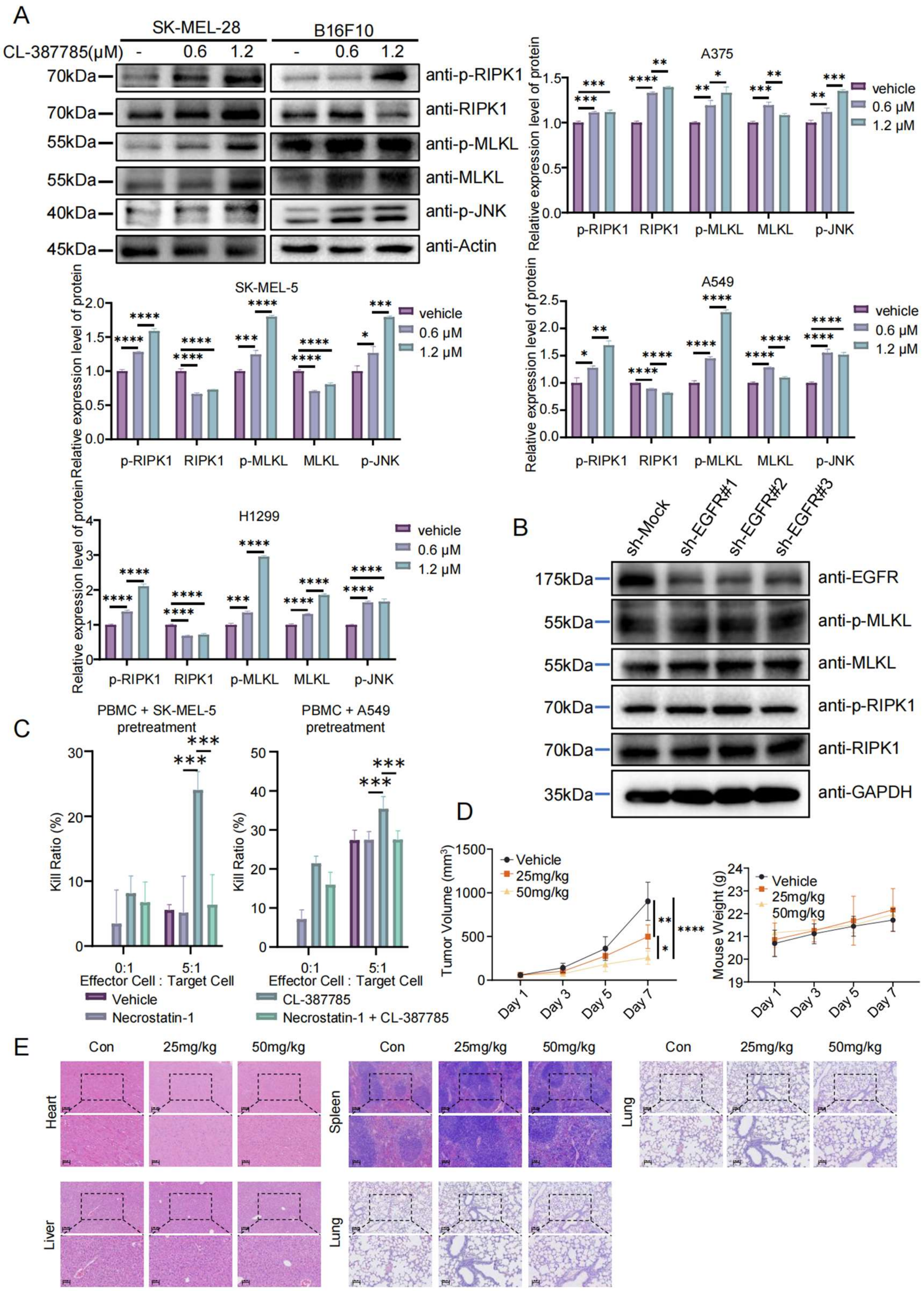
A
SK-MEL-28
B16F10
CL-387785(μM) - 0.6 1.2 - 0.6 1.2
70kDa
70kDa
55kDa
55kDa
40kDa
45kDa
anti-p-RIPK1
anti-RIPK1
anti-p-MLKL
anti-MLKL
anti-p-JNK
anti-Actin
A375
SK-MEL-5
A549
H1299
Relative expression level of protein
vehicle
0.6 μM
1.2 μM
p-RIPK1 RIPK1 p-MLKL MLKL p-JNK
B
sh-Mock
sh-EGFR#1
sh-EGFR#2
sh-EGFR#3
175kDa
55kDa
55kDa
70kDa
70kDa
35kDa
anti-EGFR
anti-p-MLKL
anti-MLKL
anti-p-RIPK1
anti-RIPK1
anti-GAPDH
C
PBMC + SK-MEL-5 pretreatment
PBMC + A549 pretreatment
Kill Ratio (%)
0:1
5:1
Effector Cell : Target Cell
Vehicle
Necrostatin-1
CL-387785
Necrostatin-1 + CL-387785
D
Tumor Volume (mm³)
Mouse Weight (g)
Vehicle
25mg/kg
50mg/kg
Day 1
Day 3
Day 5
Day 7
E
Con
25mg/kg
50mg/kg
Heart
Liver
Spleen
Lung

Fig. S4. CL-387785 induces necroptosis independent of the EGFR pathway. (A) Western blot analysis was used to assess the protein levels of phosphorylated RIPK1 (p-RIPK1), RIPK1, phosphorylated MLKL (p-MLKL), MLKL, and phosphorylated JNK (p-JNK) in SK-MEL-28 and B16F10 cell lysates. Densitometric quantification of the Western blot data is shown in the right panel. n=3. (B) Western blot analysis was used to assess the protein levels of EGFR, phosphorylated RIPK1 (p-RIPK1), RIPK1, phosphorylated MLKL (p-MLKL), MLKL, and phosphorylated JNK (p-JNK) in A375 cell lysates under EGFR knockdown conditions. (C) In vitro cytotoxicity assays were performed to assess the effect of necroptosis inhibition on the immunostimulatory effects of CL-387785 on PBMCs. n=4-6. (D) B16-F10 tumor-bearing C57BL/6 mice were treated with different concentrations of CL-387785, and the effects on tumor volume and body weight were evaluated. n=6. (E) Hematoxylin and eosin (HE) staining of mouse organs (heart, liver, spleen, lung, and kidney) following treatment with different concentrations of CL-387785. ×20 magnification and ×40 magnification. n=6.

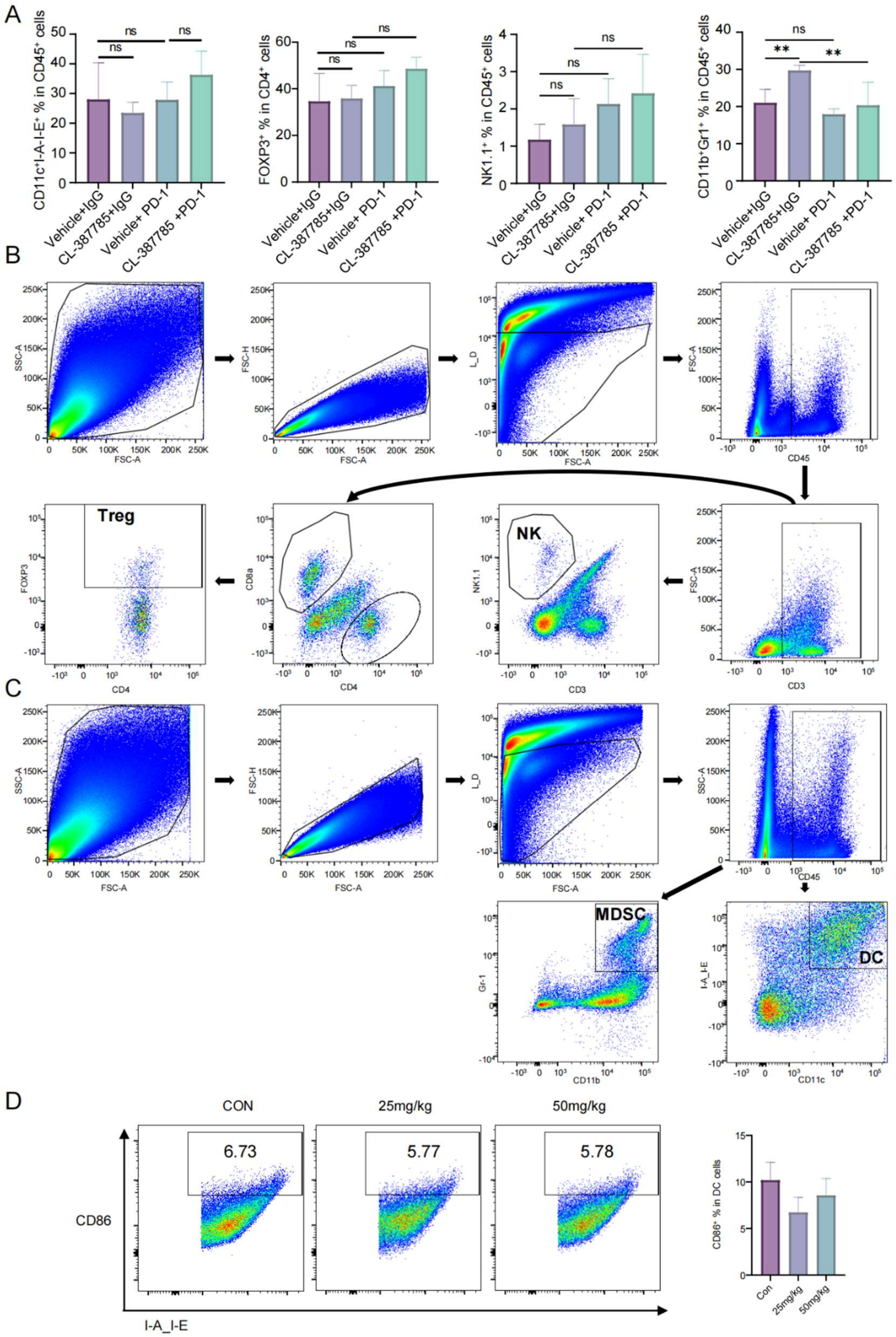
A
CD11c+I-A-I-E+ % in CD45+ cells
FOXP3+ % in CD4+ cells
NK1.1+ % in CD45+ cells
CD11b+Gr1+ % in CD45+ cells
ns
**
Vehicle+IgG
CL-387785+IgG
Vehicle+ PD-1
CL-387785 +PD-1
B
C
Treg
NK
MDSC
DC
SSC-A
FSC-A
FSC-H
L_D
CD45
CD3
CD4
CD8a
FOXP3
NK1.1
Gr-1
CD11b
I-A_I-E
CD11c
D
CON
25mg/kg
50mg/kg
6.73
5.77
5.78
CD86
I-A_I-E
CD86+ % in DC cells
Con
25mg/kg
50mg/kg

Fig. S5. CL-387785 does not affect certain immune cells in the tumor immune microenvironment. (A) Flow cytometry was used to determine the populations of $CD11c^{+}$ I-A-I-E^{+} cells, $FOXP3^{+}$ T cells, $NK1.1^{+}$ cells, and $CD11b^{+}$ $Gr1^{+}$ cells in B16-F10 tumors. n=6. (B) Flow cytometry gating strategy for Tregs and NK cells. (C) Flow cytometry gating strategy for MDSCs and DCs. (D) Flow cytometry was used to evaluate the populations of activated dendritic cells in B16F10 tumors. n=6.

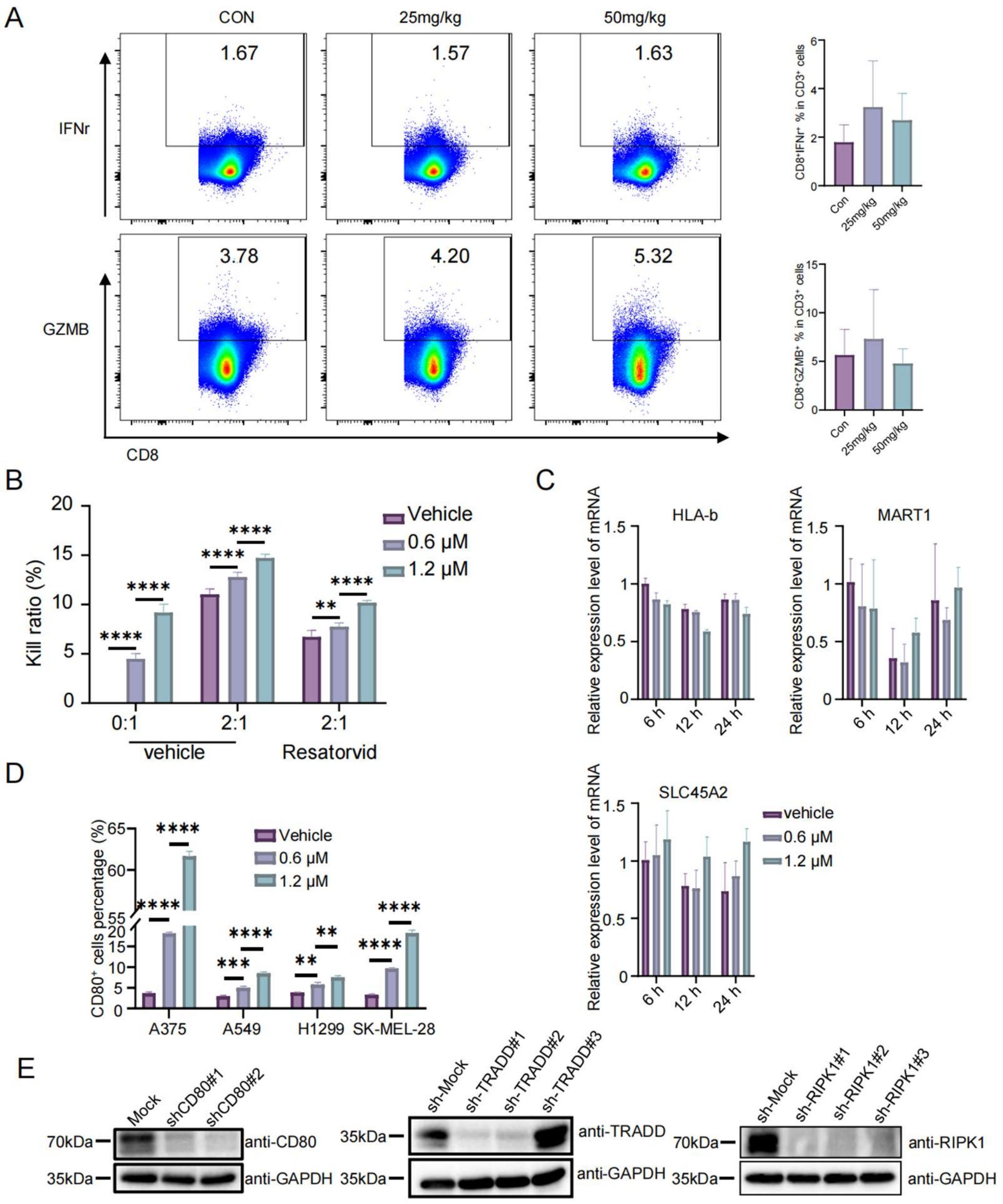
A
CON
25mg/kg
50mg/kg
1.67
1.57
1.63
IFNr
3.78
4.20
5.32
GZMB
CD8
CD8+IFNr+ % in CD3+ cells
CD8+GZMB+ % in CD3+ cells
Con
25mg/kg
50mg/kg
B
Kill ratio (%)
Vehicle
0.6 μM
1.2 μM
0:1
2:1
2:1
vehicle
Resatorvid
C
HLA-b
MART1
SLC45A2
Relative expression level of mRNA
6 h
12 h
24 h
vehicle
0.6 μM
1.2 μM
D
CD80+ cells percentage (%)
Vehicle
0.6 μM
1.2 μM
A375
A549
H1299
SK-MEL-28
E
Mock
shCD80#1
shCD80#2
70kDa
35kDa
anti-CD80
anti-GAPDH
sh-Mock
sh-TRADD#1
sh-TRADD#2
sh-TRADD#3
35kDa
anti-TRADD
anti-GAPDH
sh-Mock
sh-RIPK1#1
sh-RIPK1#2
sh-RIPK1#3
anti-RIPK1

Fig. S6. CL-387785 activates $CD8^+$ T lymphocytes by upregulating CD80 expression. (A) Flow cytometry was used to evaluate the populations of activated $CD8^+$ T lymphocytes in the spleen. n=6. (B) In vitro cytotoxicity assays were performed to assess the effect of TLR4 inhibition on the immunostimulatory effects of CL-387785 on $CD8^+$ T lymphocytes. n=6. (C) RT–PCR was used to analyze the expression levels of HLA-b, MART1 and SLC45A2 in SK-MEL-5 cells after treatment with CL-387785. n=3. (D) Flow cytometry was used to detect the percentage of $CD80^+$ cells after CL-387785 treatment. n=3. (E) Western blot analysis was used to validate the knockdown efficiency of CD80, TRADD, and RIPK1 in the A375 cell line.

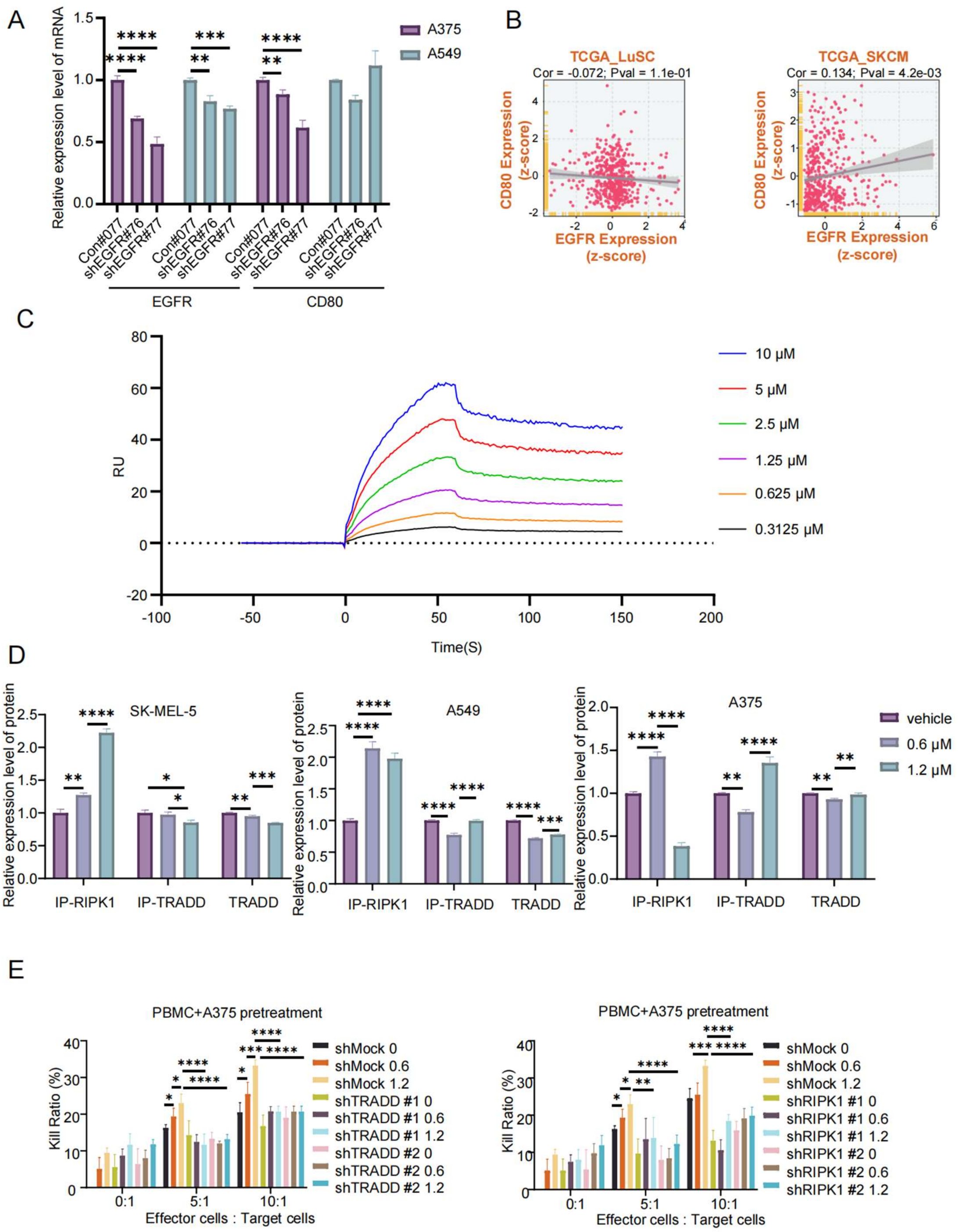

A
Relative expression level of mRNA
A375
A549
Con#077
shEGFR#76
shEGFR#77
EGFR
CD80
B
TCGA_LuSC
Cor = -0.072; Pval = 1.1e-01
TCGA_SKCM
Cor = 0.134; Pval = 4.2e-03
CD80 Expression (z-score)
EGFR Expression (z-score)
C
RU
Time(S)
10 μM
5 μM
2.5 μM
1.25 μM
0.625 μM
0.3125 μM
D
Relative expression level of protein
SK-MEL-5
A549
A375
IP-RIPK1
IP-TRADD
TRADD
vehicle
0.6 μM
1.2 μM
E
PBMC+A375 pretreatment
Kill Ratio (%)
Effector cells : Target cells
shMock 0
shMock 0.6
shMock 1.2
shTRADD #1 0
shTRADD #1 0.6
shTRADD #1 1.2
shTRADD #2 0
shTRADD #2 0.6
shTRADD #2 1.2
shRIPK1 #1 0
shRIPK1 #1 0.6
shRIPK1 #1 1.2
shRIPK1 #2 0
shRIPK1 #2 0.6
shRIPK1 #2 1.2

Fig. S7. CL-387785 targets TRADD to regulate the antitumor immune response. (A) RT–PCR was used to assess the efficiency of EGFR knockdown and the expression of CD80 in A375 and A549 cells. n=3-4. (B) The co-expression of EGFR and CD80 in melanoma and lung cancer patients was analyzed using the TCGA database. (C) SPR was used to assess the affinity between CL-387785 and TRADD. (D) Densitometric quantification of the Western blot data shown in Figure 5G. n=3. (E) In vitro cytotoxicity assays were performed to assess the effects of TRADD and RIPK1 knockdown on the immunostimulatory effects of CL-387785 on PBMCs. n=6.

A

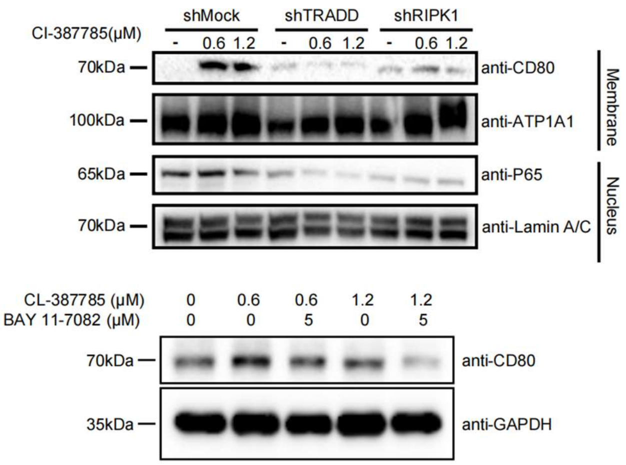

B

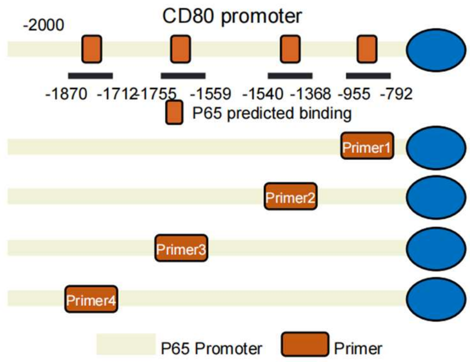

Fig. S8. CL-387785 upregulates CD80 by promoting the nuclear translocation of p65. (A) Western blotting was used to assess the effects of TRADD and RIPK1 knockdown on CD80 and P65 expression after treatment with CL-387785. (B) A schematic diagram predicting the binding sites of P65 on the CD80 promoter.

Table S1. A list of primers used for qPCR

Gene	Species		Primer sequence
CD80	Homo Sapiens	Forward	AAACTCGCATCTACTGGCAAA
		Reverse	GGTTCTTGTACTCGGGCCATA
CD80	Mice	Forward	ACCCCCAACATAACTGAGTCT
		Reverse	TTCCAACCAAGAGAAGCGAGG
HLA-b	Homo Sapiens	Forward	GATGGCGAGGACCAAACTCA
		Reverse	CTCCGATGACCACAACTGCT
MART1	Homo Sapiens	Forward	GCTCACTTCATCTATGGTTACCC
		Reverse	GACTCCCAGGATCACTGTCAG
SLC45A2	Homo Sapiens	Forward	CTGGCCGCCACATCTATAAAT
		Reverse	GTAGCAGAACTCTCTTCCGAAC

Table S2. A list of primers for used ChIP-qPCR.

Gene		Primer sequence
CD80 Primer1	Forward	GTACATAATCTTGGAAGAG
	Reverse	CCTGCCTTGGAGAAACAC
CD80 Primer2	Forward	GCGCTAATGGCAAAGTTG
	Reverse	GATCCACCCTCCTCGGCCTC
CD80 Primer3	Forward	GACCAGCCTGGCCAATATG
	Reverse	GCTGGAGTGCAGTGGTGC
CD80 Primer4	Forward	CAGTGGAGTCCTTTCATAG
	Reverse	GCAAACCGTGTGGGTGAG
